# Sarcomas in the United States: Recent trends and a call for improved staging

**DOI:** 10.18632/oncotarget.26809

**Published:** 2019-03-29

**Authors:** Michele M. Gage, Neeraja Nagarajan, Jessica M. Ruck, Joseph K. Canner, Salma Khan, Katherine Giuliano, Faiz Gani, Christopher Wolfgang, Fabian M. Johnston, Nita Ahuja

**Affiliations:** ^1^ Department of Surgery, Johns Hopkins University, Baltimore, Maryland, USA; ^2^ Rehman Medical Institute, Hayatabad, Pakistan; ^3^ Department of Surgery, Yale University, New Haven, Connecticut, USA

**Keywords:** sarcoma, mesenchymal tumors, connective tissue tumors, SEER, trends of sarcoma

## Abstract

**Background and objectives:**

Sarcomas represent a heterogeneous group of tumors, and there is lack of data describing contemporary changes in patterns of care. We evaluated the epidemiology of sarcomas over 12 recent years

**Methods:**

The Surveillance, Epidemiology and End Results (SEER) database was queried for sarcoma cases from 2002-2014. Patient, tumor and treatment factors, and trends over time were studied overall and by subtype. Univariable and multivariable logistic regression models and 5-year survival and cause-specific mortality (CSM) were summarized.

**Results:**

There were 78,527 cases of sarcomas with an overall incidence of 7.1 cases per 100,000 people, increasing from 6.8 in 2002 to 7.7 in 2014. Sarcoma NOS(14.8%) and soft tissue(43.4%) were the most common histology and primary site, respectively. A majority of tumors were high-grade(33.6%) and >5 cm(51.3%). CSM was 28.6% and 5-year survival was 71.4%. Many patients had unknown-grade(42.2%), which associated with 2.6 times increased odds of no surgical intervention.

**Conclusions:**

This comprehensive national study highlights important trends including increasing incidence, changing histologic types, and underestimation of true incidence. A large proportion of sarcomas are inadequately staged (unknown-grade 42.2%) with lack of appropriate surgical treatment. Our study highlights need for standardization of care for sarcomas.

## INTRODUCTION

Sarcomas are a heterogeneous group of over 80 different tumors arising from mesenchymal or connective tissue. In 2018, soft tissue sarcomas will represent approximately 0.8% of all cancers in the United States (US)and are among the top five causes of cancer deaths for those under 20 years old [1]. It is estimated that approximately 13-16,000 new cases and 5-6,000 deaths will be attributable to sarcomas in the US [1, 2].

The variability of all subtypes of sarcomas are not well described due to the heterogeneity of the disease, with subtypes varying in biology, behavior, and treatment responses [3–6]. The complexity and rarity of sarcomas make them challenging to study as well as medically manage. This has driven the development of many long-term institutional, multi-institutional, and national databases that collect epidemiological and clinical data on sarcomas to better understand the disease processes [3–6]. This study utilizes a nationally representative cancer database, the Surveillance, Epidemiology and End Results (SEER), to study sarcomas in the US over 12 recent years and evaluate trends in epidemiology, management, and survival.

## RESULTS

### Trends of sarcomas over time

**Figure 1:**
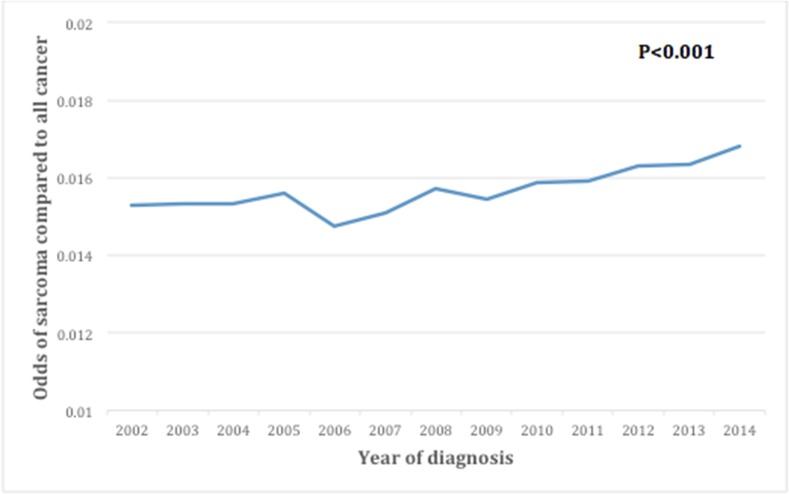
Trends over time for incidence of sarcomas

**Figure 2:**
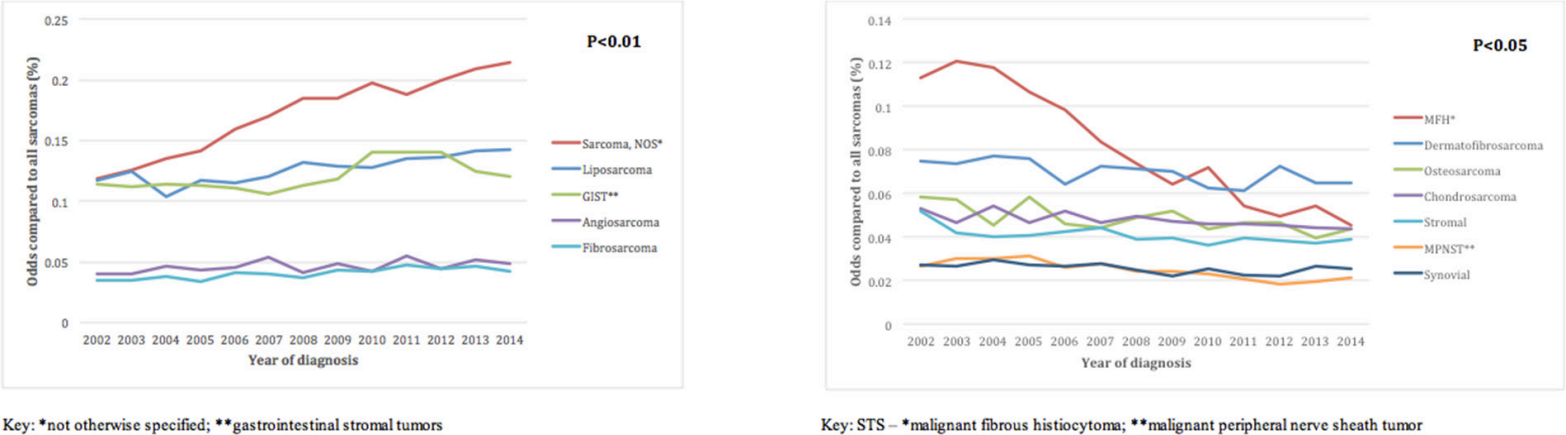
**(A)** Trends over time for sarcoma subtypes that are increasing in incidence. **(B)** Trends over time for sarcoma subtypes that are decreasing in incidence.

A total of 78,527 patients with sarcomas were identified in SEER from 2002-2014. Overall age-adjusted incidence rate of sarcomas in this period was 7.1/100,000 individuals. From 2002-2014, the incidence increased from 6.8 to 7.7, andthe odds of sarcomas compared to all cancers increased from 0.015 to 0.017 (p<0.001) (Figure 1). Compared to all sarcomas, the odds of being diagnosed with sarcoma-not-otherwise-specified (NOS) increased the most (Figure 2A), while malignant fibrous histiocytoma (MFH) decreased the most (Figure 2B).

### Epidemiology and characteristics of sarcomas

The median age at diagnosis was 58 years (interquartile range, IQR: 43-72) with approximately half of the patients being female (50.6%), a majority of patients being White (78.1%) and a majority of patients living in metropolitan areas (89.2%, Table 1). The most common histology was sarcoma-NOS (14.8%), followed by leiomyosarcoma (14.6%). A third of tumors were grade III/IV (38.0%), while the majority were of unknown grade (42.2%). Approximately half of sarcomas were over 5 cm (51.3%) and a quarter were unknown (24.5%). A majority of patients did not have spread to the lymph nodes (80.3%) or distant metastasis (75.8%) at diagnosis. Surgical resection was performed in most patients (79.7%), with less undergoing radiation therapy (26.0%).

**Table 1:** Characteristics of sarcoma patients (n=78,527)

	No. of patients (%) (n=78,527)	5-year cause-specific survival (%)	p-value^&^
**Age**	58 (43-72)	NA	NA
**Sex**			0.004
Female	39,720 (50.6)	71.0	
Male	38,807 (49.4)	70.3	
**Race**			<0.001
White	61,344 (78.1)	71.9	
Black	9,831 (12.5)	67.2	
Others	7,352 (9.4)	72.3	
**Location**			<0.001
Rural	900 (1.1)	68.9	
Urban	7,575 (9.7)	70.0	
Metropolitan	69,957 (89.2)	71.6	
**Histology**			<0.001
Leiomyosarcoma	11,487 (14.6)	60.5	
MFH*	5,707 (7.3)	77.0	
Liposarcoma	8,855 (11.3)	82.8	
Dermatofibroma	5,093 (6.5)	99.2	
Rhabdomyosarcoma	2,581 (3.3)	54.7	
Angiosarcoma	3,471 (4.4)	53.8	
GIST**	8,486 (10.8)	80.4	
Fibrosarcoma	3,061 (3.9)	82.9	
Sarcoma, NOS***	11,581 (14.8)	55.2	
Osteosarcoma	3,603 (4.6)	65.2	
Chondrosarcoma	3,577 (4.6)	81.9	
Synovial	1,955 (2.5)	65.6	
Stromal	3,058 (3.9)	75.6	
MPNST****	1,879 (2.4)	65.4	
Ewing sarcoma	1,828 (2.3)	64.0	
Other^	2,305 (2.9)	70.4	
**Primary Site**			<0.001
Soft tissue	34,064 (43.4)	71.3	
Abdominal viscera	19,533 (24.9)	68.1	
Thoracic viscera	2,588 (3.3)	56.8	
RPS+	3,511 (4.5)	58.6	
Head and Neck	1,816 (2.3)	73.9	
Bone	8,583 (10.9)	70.6	
Other#	8,432 (10.7)	89.2	
**Primary Grade**^**A**^			<0.001
Grade I	7,808 (9.9)	87.5	
Grade II	7,779 (9.9)	79.4	
Grade III & IV	29,830 (38.0)	51.9	
Unknown	33,110 (42.2)	68.4	
**Primary Size**			<0.001
<5 cm	19,017 (24.2)	88.1	
More than 5cm	40,292 (51.3)	65.3	
Unknown/Not found	19,218 (24.5)	67.0	
**Local Extension**			<0.001
Confined to site of origin	24,480 (31.2)	83.5	
No primary	1 (0.0)	NA	
Localized	15,340 (19.5)	78.3	
Adjacent connective tissue	16,657 (21.2)	75.7	
Adjacent organs/structures	11,684 (14.9)	45.1	
Unknown	10,365 (13.2)	52.0	
**Lymph node**			<0.001
No	60,063 (80.3)	75.9	
Yes	3,955 (5.0)	35.1	
Unknown	11,509 (14.7)	57.0	
**Metastasis at diagnosis**			<0.001
No	59,524 (75.8)	79.2	
Yes	12,805 (16.3)	30.1	
Unknown	6,198 (7.9)	74.9	
**Surgery on Primary**			<0.001
Yes	62,583 (79.7)	77.6	
No	15,358 (19.6)	41.8	
Unknown	586 (0.8)	60.6	
**Radiation**			<0.001
Yes	20,408 (26.0)	66.0	
No	57,285 (73.0)	73.5	
Unknown	834 (1.0)	64.9	

Key: ^*^malignant fibrous histiocytoma; ^**^gastrointestinal stromal tumors; ^***^not otherwise specified; ^****^malignant peripheral nerve sheath tumor; ^^^including malignant mesochymoma, odontogenic tumor, clear cell sarcoma, myxosarcoma, malignant hemangiopericytoma, malignant giant cell tumor, malignant granular cell tumor, alveolar soft part sarcoma, and desmoplastic small round cell tumor; ^A^not including GIST; ^+^retroperitoneal sarcoma; ^#^Other in primary site includes miscellaneous, other endocrine organs, non-epithelial skin; ^&^from log-rank test

### Patient, clinicopathologic, and treatment characteristics by histology

**Figure 3:**
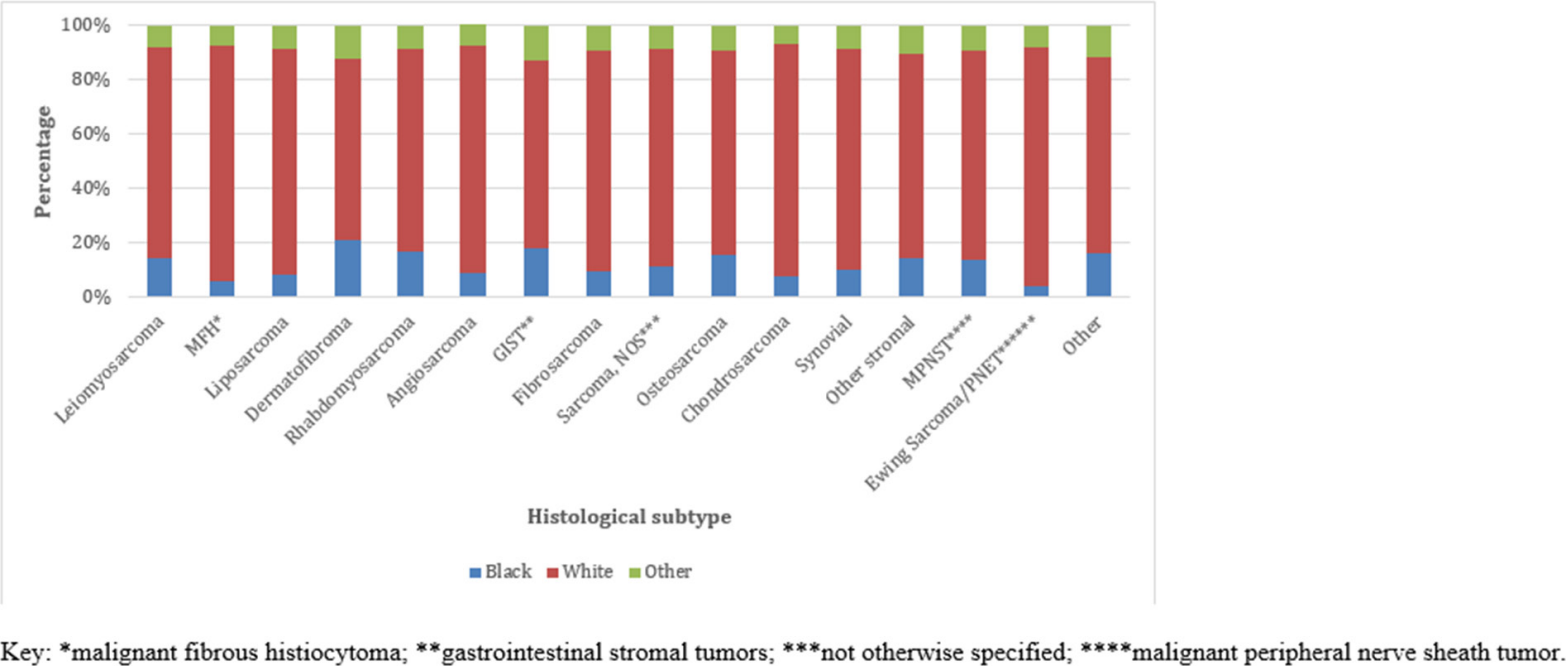
Racial distribution by histological subtype (n=64,645)

The epidemiology and characteristics of sarcomas were different across histological subtypes (Table 2, Figure 3). The median age at diagnosis varied from 17 years in rhabdomyosarcoma to 72 years in MFH. Females were a large majority of those diagnosed with stromal tumors (98.2%) but were a minority in MFH (33.6%).

**Table 2:** Characteristics of sarcoma by histology (n=78,527)

Histology	Age (median, IQR)	Female (%)	Grade III/IV (%)	Confined to SO^&^ (%)	LN^$^ involvement (%)	Mets at diagnosis (%)	Surgery on primary (%)	Radiation (%)
Leiomyosarcoma	60 (49-72)	66.7	39.4	35.5	4.4	21.0	82.9	23.7
MFH^*^	72 (60-81)	33.6	44.5	33.8	2.9	8.2	84.6	34.6
Liposarcoma	61 (49-72)	39.2	26.5	37.9	1.6	6.2	90.5	32.3
Dermatofibroma	43 (32-54)	53.9	1.6	25.5	0.3	0.8	93.9	6.0
Rhabdomyosarcoma	17 (6-54)	45.2	100	21.9	23.6	29.5	57.2	54.4
Angiosarcoma	68 (54-79)	54.4	35.1	30.4	7.6	19.8	66.0	25.7
GIST^**^	64 (54-74)	47.7	NA	25.0	4.3	19.9	77.9	0.8
Fibrosarcoma	60 (46-73)	48.3	33.0	42.1	2.1	7.1	90.2	38.3
Sarcoma, NOS^***^	65 (51-77)	47.3	57.4	26.6	8.1	20.7	63.7	35.4
Osteosarcoma	22 (14-49)	46.0	62.8	19.0	2.8	12.7	81.4	10.5
Chondrosarcoma	53 (41-67)	43.8	18.5	28.5	1.6	8.3	84.7	17.0
Synovial	39 (25-54)	46.9	100	36.8	4.7	16.1	83.8	53.1
Stromal	53 (45-65)	98.2	28.8	54.4	6.7	14.5	91.0	19.1
MPNST^****^	48 (32-63)	45.5	38.7	31.6	4.3	13.6	83.5	41.7
Ewing sarcoma	18 (12-28)	41.4	100	15.9	6.8	24.5	62.5	44.1
Other*^*	45 (26-61)	48.6	24.2	31.9	10.0	20.3	79.8	30.3

Key: malignant fibrous histiocytoma; ^**^gastrointestinal stromal tumors; ^***^not otherwise specified; ^****^malignant peripheral nerve sheath tumor; ^^^including malignant mesochymoma, odontogenic tumor, clear cell sarcoma, myxosarcoma, malignant hemangiopericytoma, malignant giant cell tumor, malignant granular cell tumor, alveolar soft part sarcoma, and desmoplastic small round cell tumor; ^&^site of origin; ^$^Lymph node

All p<0.001

**Figure 4:**
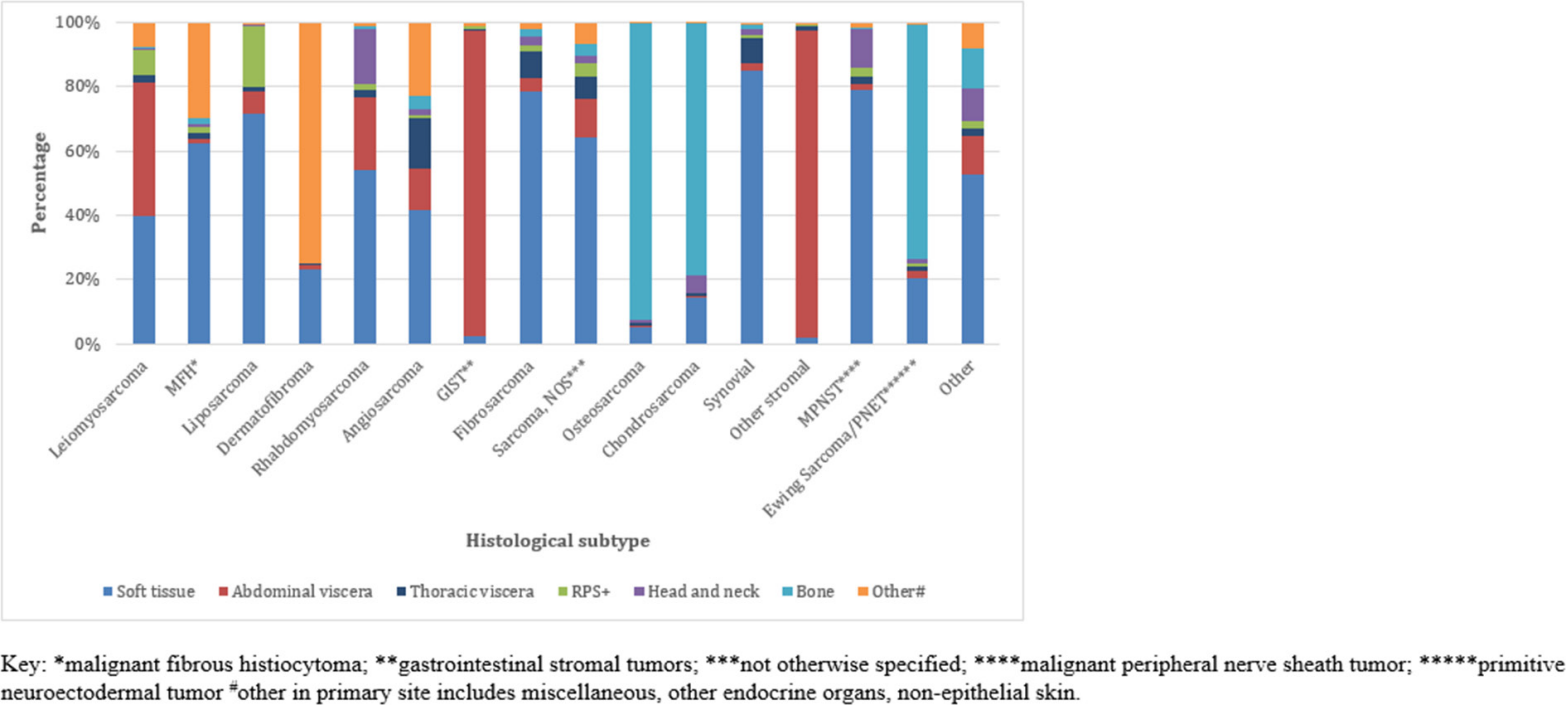
Primary anatomical site across histological subtype (n=64,645)

Primary site varied widely across histological subtypes (Figure 4). Abdominal viscera was the most common site for gastrointestinal stromal tumors (GIST) (95.7%) and stromal tumors (97.9%). Bone was the most frequent site for osteosarcoma (92.3%) and chondrosarcoma (78.6%). Other primary site, which includes skin, was the most common site for dermatofibrosarcoma (74.8%). Soft tissue was the most frequent site for the other histological subtypes (p<0.001).

In addition to the inherently high-grade histologic subtypes, osteosarcomas had the highest (62.8%) proportion of tumors that were grade III/IV. Lymph node involvement and metastasis were highest in rhabdomyosarcoma (23.6%). Surgery was performed on almost all patients with dermatofibrosarcoma (93.9%) and in a lesser proportion of patients with rhabdomyosarcoma (57.2%). Conversely, radiation therapy was most common in rhabdomyosarcoma (54.4%) (Table 2).

### Survival in sarcomas

**Figure 5:**
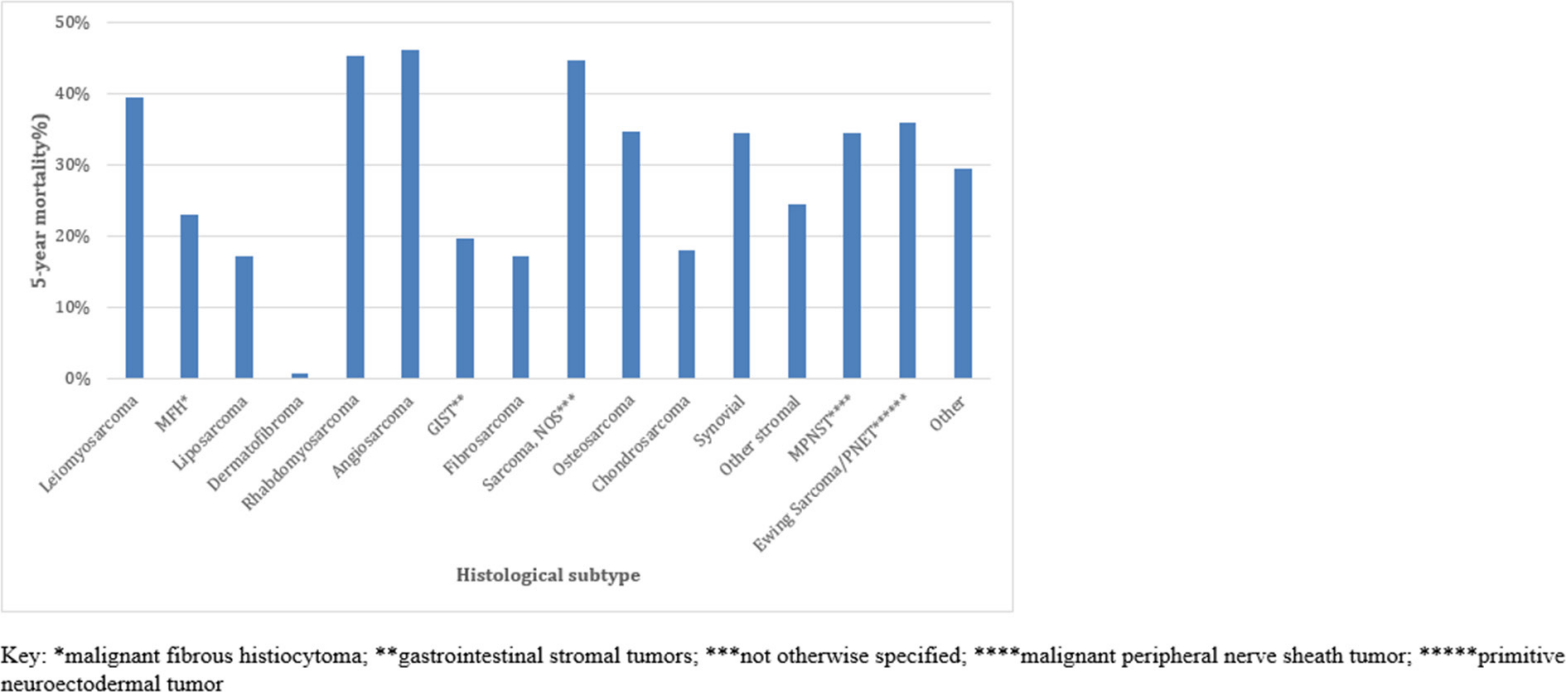
Cause-specific mortality by histological subtype (n=64,645)

Overall, 5-year cause specific mortality (CSM) was 28.6% while 5-year all-cause mortality (ACM) was 34.7%. In this cohort, the 1, 5, and 10-year survival were 87.0%, 71.4%, and 65.5%, respectively. CSM was different across histological subtypes, with the highest CSM in angiosarcoma (52.8%) and the lowest in dermatofibrosarcoma (0.1%) (Figure 5).

Five-year survival also differed significantly across other variables (Table 1). Among demographic variables, a metropolitan location was associated with higher 5-year survival (all p<0.01). Grade of the tumor showed significant differences in 5-year survival: 87.5% for grade I, 79.4% for grade II, and 51.9% for grade III/IV and 68.4% for unknown grade (p<0.001). Among treatment factors, patients who underwent surgery and those who did not receive radiation had higher 5-year survival (all p<0.001).

Further analyses comparing 5-year survival of surgery in localized disease compared to metastatic disease sarcoma cases were performed. There were 59,524 patients with primary, or localized disease, and 12,805 patients with metastatic disease. Of patients with localized disease, 85.6% underwent surgery, while 13.6% did not, for unknown reasons. Of these patients, the 5-year survival was 76.8% in the surgery group, and less than 50% in the non-operative group at 48.8% (p<0.001). Of patients with metastatic disease, 49.6% underwent surgery, while 50% did not. Of these patients, the 5-year survival was 36.3% in the surgery group, and 16% in the non-operative group (p<0.001).

### Unknown grade

Given the substantial proportion of patients with unknown grade, a subset analysis (n=58,584) was performed to better understand the characteristics associated with unknown grade. Grade was unknown in 34.2% of patients within this cohort.

Patient, tumor and treatment factors varied between those with unknown grade and those with a known grade (Table 3); the patients with unknown grade were more likely to be older (62 vs. 59, p<0.001), Black (11.7% vs. 10.9%, p=0.013), and not undergo surgery (31.0% vs. 13.2%, p<0.001). On multivariable logistic regression, unknown grade was associated with a 2.6 times and 1.5 times increased odds of not receiving surgery and radiation, respectively (Table 4).

**Table 3:** Comparison of characteristics between known and unknown grade (n=58,584)

Variable	Unknown grade (n=20,025) (%)	Known grade (n=38,559) (%)	p-value
**Age (median, IQR)**	62 (47-76)	59 (46-72)	<0.001
**Sex**			<0.001
Female	50.4	51.8	
Male	49.6	48.2	
**Race**			<0.001
White	76.5	80.5	
Black	11.7	10.9	
Others	8.8	8.6	
**Histology**			<0.001
Leiomyosarcoma	22.8	17.9	
MFH*	13.6	7.7	
Liposarcoma	7.1	19.3	
Angiosarcoma	9.0	4.4	
Fibrosarcoma	4.2	5.8	
Sarcoma, NOS***	19.2	20.1	
Osteosarcoma	5.0	6.8	
Chondrosarcoma	3.4	7.5	
Stromal	4.4	5.6	
MPNST****	4.1	2.7	
Other^	7.3	2.2	
**Primary Site**			<0.001
Soft tissue	42.9	53.5	
Abdominal viscera	20.1	17.3	
Thoracic viscera	4.5	3.6	
RPS+	3.9	6.7	
Head and Neck	2.9	1.9	
Bone	9.2	13.9	
Other#	16.6	3.0	
**Primary Size**			<0.001
<5 cm	22.0	21.9	
More than 5cm	36.3	62.3	
Unknown/Not found	41.7	15.8	
**Local Extension**			<0.001
Confined to site of origin	27.9	36.1	
Localized	15.8	21.1	
Adjacent connective tissue	18.8	19.0	
Adjacent organs/structures	13.4	16.3	
Unknown	24.1	7.5	
**Lymph node**			
No	69.3	86.2	<0.001
Yes	5.3	4.4	
Unknown	35.6	9.3	
**Metastasis at diagnosis**			
No	59.2	72.9	<0.001
Yes	17.4	13.4	
Unknown	23.5	13.7	
**Surgery on Primary**			
Yes	69.0	86.8	<0.001
No	31.0	13.2	
**Radiation**			
Yes	78.7	65.7	<0.001
No	20.4	32.9	
Unknown	0.9	1.4	

Key: malignant fibrous histiocytoma; ^**^gastrointestinal stromal tumors; ^***^not otherwise specified; ^****^malignant peripheral nerve sheath tumor; ^^^including malignant mesochymoma, odontogenic tumor, clear cell sarcoma, myxosarcoma, malignant hemangiopericytoma, malignant giant cell tumor, malignant granular cell tumor, alveolar soft part sarcoma, and desmoplastic small round cell tumor.

**Table 4:** Multivariable logistic regression for the odds of having unknown grade (n=58,086)

Variable	OR (95% CI)	p-value
**Age**	0.997 (0.996-0.998)	<0.001
**Sex**		
Female	Ref	
Male	1.02 (0.98-1.07)	0.304
**Race**		
White	Ref	
Black	1.08 (1.02-1.15)	0.015
Other	0.98 (0.92-1.05)	0.614
**Histology**		
Leiomyosarcoma	Ref	
MFH*	1.20 (1.12-1.31)	<0.001
Liposarcoma	0.43 (0.39-0.46)	<0.001
Angiosarcoma	1.02 (0.93-1.15)	0.655
Fibrosarcoma	0.77 (0.69-0.84)	<0.001
Sarcoma, NOS***	0.58 (0.55-0.62)	<0.001
Osteosarcoma	0.80 (0.70-0.92)	0.001
Chondrosarcoma	0.50 (0.44-0.57)	<0.001
Stromal	0.52 (0.47-0.58)	<0.001
MPNST****	1.49 (1.33-1.67)	<0.001
Other^	2.87 (2.58-3.18)	<0.001
**Primary Site**		
Soft tissue	Ref	
Abdominal viscera	1.48 (1.39-1.58)	<0.001
Thoracic viscera	1.31 (1.19-1.44)	<0.001
RPS+	0.92 (0.84-1.02)	0.105
Head and Neck	1.19 (1.05-1.37)	0.008
Bone	0.76 (0.68-0.85)	<0.001
Other#	4.07 (3.74-4.43)	<0.001
**Primary Size**		
<5 cm	Ref	
More than 5cm	0.72 (0.68-0.76)	<0.001
Unknown/Not found	1.65 (1.56-1.75)	<0.001
**Local Extension**		
Confined to site of origin	Ref	
Localized	1.10 (1.04-1.16)	0.001
Adjacent connective tissue	0.97 (0.91-1.03)	0.285
Adjacent organs/structures	0.91 (0.85-0.97)	0.003
Unknown	1.49 (1.39-1.61)	<0.001
**Lymph node**		
No	Ref	
Yes	1.04 (0.95-1.14)	0.367
Unknown	1.41 (1.31-1.50)	<0.001
**Metastasis at diagnosis**		
No	Ref	
Yes	0.91 (0.85-0.97)	0.002
Unknown	0.97 (0.91-1.03)	0.302
**Surgery on Primary**		
Yes	Ref	
No	2.59 (2.45-2.73)	<0.001
**Radiation**		
Yes	Ref	
No	1.47 (1.40-1.54)	<0.001
Unknown	1.00 (0.83-1.21)	0.961

Key: ^*^malignant fibrous histiocytoma; ^**^gastrointestinal stromal tumors; ^***^not otherwise specified; ^****^malignant peripheral nerve sheath tumor; ^^^including malignant mesochymoma, odontogenic tumor, clear cell sarcoma, myxosarcoma, malignant hemangiopericytoma, malignant giant cell tumor, malignant granular cell tumor, alveolar soft part sarcoma, and desmoplastic small round cell tumor; OR: Odds Ratio.

## DISCUSSION

This nationwide study of approximately 78,000 patients over 12 recent years demonstrates an increase in the incidence of sarcomas, summarizes the salient epidemiological features of sarcomas and their subtypes, documents survival outcomes, and identifies the significance of a diagnosis of unknown tumor grade. For the year 2014, we identified 6,888 sarcomas patients in a cohort representing28% of the US population, while the national estimate for primary cancers of the soft tissue based on SEER was approximately 12,000 patients [2]. Based on this study, the national estimates for new cases may potentially underestimate the true incidence of sarcomas, of 24,000, by 50%. This underestimation is partially the result of national estimates calculated for sarcoma arising only from soft tissue, whereas this study included all sarcomas based on International Classification for Oncology, 3^rd^ edition (ICD-O-3) histology codes defined by the World Health Organization (WHO) [7]. Toro el al., among others, have shown that there is a national underestimation of true sarcoma incidence due to exclusion of sarcomas that arise from organs [4]. This underestimation results in underrepresentation of visceral sarcomas in the epidemiology of sarcomas nationally, and ultimately affects survival and treatment estimates.

In addition to this underestimation, there has been a steady increase in the overall incidence of sarcomas in the US from 2002-2014. Sarcoma-NOS has nearly doubled in incidence. The emergence of new subtypes of sarcomas, such as fibroxyoid sarcoma and sclerosing epithelioid fibrosarcoma, as well as the reclassification of other important subtypes, have led to proportional differences within sarcomas and are also a potential cause for the increasing incidence [4, 7]. Notably, the incidence of sarcoma rapidly increases after age 50, and the increased population of older individuals is likely contributing to increasing incidence [8]. All 5 histological subtypes of sarcomas that had a significantly increased trend over time – GIST, liposarcoma, angiosarcoma, fibrosarcoma and sarcoma-NOS – predominantly affect individuals over 50 years of age.

Survival outcomes were also different across patient, tumor and treatment variables. The 5-year survival was 71.4%, which falls within the range reported in the literature and represents an increase in survival over the last decade due to improved diagnostic and therapeutic measures [9]. Tumors with higher grade, increased size, local extension, and metastasis all exhibited lower survival, as expected [10, 11]. Surgical resection is the only curative treatment for sarcoma, and surgical patients had significantly higher survival [3, 12]. However, Black patients and patients from rural areas showed lower 5-year survival when compared to Whites and patients from metropolitan areas, respectively. Further risk-adjusted histology-specific studies are mandated to understand if these differences represent a disparity in access to care among minorities and under-served populations or are a result of differences in histological make-up and tumor behavior in these populations [1, 4, 11].

Most striking in our study was the proportion of patients with unknown grade, as well as the increase in sarcoma-NOS diagnoses. Appropriate grading and histological analysis is a crucial part of the diagnostic workup for sarcomas, as they are the most important risk factors for a number of patient outcomes. Grade has been demonstrated to be an important risk factor disease-free survival, recurrence-free survival, local recurrence, and presence of distant metastasis [12]. SEER has a substantial percentage of patients with missing/unknown grade. Several earlier STS studies using SEER have shown similar rates of unknown grade, extending up to 50% [4, 13]. Notably, our study shows that the incidence of tumors with unknown grade has remained stable through years included in the study, highlighting that the reasons for high rates of unknown grade have not been addressed. SEER data is extensively audited for completeness and accuracy and generally has little missing information in contrast to administrative databases [14], therefore, missing data could potentially represent inadequate workup at the hospital level as well as potential under-treatment due to refrain from surgery. The increase in sarcoma-NOS diagnoses is troublesome for potentially inadequate histologic workup, as well.

Early studies using SEER inferred unknown grade to be a proportional mixture of patients of other grades [13]; we investigated specific characteristics that are associated with an unknown grade and found that important factors associated with increased odds of having an unknown grade are tumors that belong to the “other” histological subtype and “other” primary site. The “other” histological subtype consists of over 20 extremely rare tumors, each making up less than 0.5% of STS. These highly rare tumors require multi-disciplinary expertise to diagnose and grade [3]. Further, tumors with unknown grade were more likely to have unknown size, local extension, and lymph node status. Earlier studies have suggested that one of the reasons for the high proportion of unknown grade for STS in large national databases may be because those tumors did not require grade to guide treatment decisions [13]. However, this study shows that patients with a tumor of unknown grade are potentially under-treated with a 2.6 times and 1.5 times increased odds of not undergoing surgical resection and radiation after accounting for a variety of risk factors. These findings suggest that unknown grade tumors may not, in fact, be a proportional mixture of other grades but represent disproportionately rare subtypes of sarcoma tumors that are being inadequately graded and subsequently, possibly inadequately managed.

In contrast to the findings in this study, single high volume institutional studies from centers that specialize in sarcoma care have almost no unknown grade or size in their workup of patients with sarcomas; these high volume institutions also achieve higher rates of therapy and improved outcomes [3, 15]. Effective management of sarcomas depend on accurate grading and staging to guide treatment strategies. There has been extensive research that has shown that patient outcomes are improved when sarcomas are treated by multi-disciplinary teams in specialized high-volume sarcoma centers[5, 16, 17]. Despite these findings, the current study suggests that there is still under-triage and under-treatment of sarcoma patients.

The study has several limitations, including those related to the SEER database. SEER does not collect data on patient comorbidities, local recurrence, or surgical margins. Grouping of histological types meant that we did not offer comments on the over 80 subtypes of sarcomas, however, the grouping assisted presenting overall epidemiological data on sarcomas. We were unable to comment on the predictors of survival in patients with STS due to the heterogeneity of the data. Despite these limitations, this study provides a comprehensive update on the epidemiology of sarcomas.

## MATERIALS AND METHODS

### Study population and variables

A retrospective study was performed utilizing SEER Program data from 2002-2014. The SEER database collects data from 20 registries representing 28% of the US population, capturing information on 98% of incident cancers in regions where data is collected [14, 18]. All patients with a diagnosis of sarcoma were identified using the ICD-O-3 histology codes [7] as defined by the 2013 WHO criteria [19]. Histologies and primary site categories are listed in Table 1. Excluded histologies are listed in the Appendix.

Histological grade for sarcoma is reported in the SEER database using a three-tier system of low, medium, and high plus unknown/missing grade [20], however, the preferred grading system for sarcomas is the French Federation of Cancer Centers Sarcoma Group (FNCLCC), a four-tier system plus the unknown category [21]. The data was re-coded in accordance with FNCLCC, with grade III and IV substituting for the high-grade group in SEER, which is also consistent with the American Joint Commission on Cancer (AJCC) staging [10]. In SEER, the majority of rhabdomyosarcoma, synovial sarcoma, and Ewing sarcoma are coded as unknown/missing grade. These subtypes were re-coded as grade III/IV as these subtypes are inherently high-grade [3]. The majority of dermatofibrosarcoma cases also had an unknown/missing grade, however, this was kept as is, as there are reports of high-grade variants [22]. Grade was left undefined in GIST, as mitotic index was not reported.

Demographic variables such as age, sex, race, and location were also analyzed. In addition to histology, site, and grade, tumor-specific factors such as size, local extension, lymph node status, and distant metastasis at diagnosis and treatment variables, such as surgery and radiation, were included. Survival variables such as CSM and ACM were also included; SEER confirms deaths by death certificates [20].

### Statistical analysis

Age-adjusted incidence rates were calculated using data from the 2000 and 2010 U.S. census [21]. Score test for trends of odds was performed to study changes in the incidence over time. Incidences of sarcoma versus all cancers in SEER and trends for each histological subtype were also compared.

For descriptive analysis, continuous variables were summarized by mean/standard deviation and median/IQR for normally and non-normally distributed variables, respectively. Student's t-test and Kruskal-Wallis test were used for comparing normal and non-normal continuous variables, respectively. Categorical variables were described using counts/proportions and compared using Pearson's chi-square test. Overall and variable-specific 5-year and median survival were compared using the log rank test. The event of interest for the Kaplan-Meier survival analysis was CSM.

As grade is a part of the AJCC staging criteria [10] and impacts prognosis, a subset analysis was performed to better determine the characteristics of tumors with unknown grade. The subset analyses excluded the following histological subtypes - GIST, dermatofibrosarcoma, rhabdomyosarcoma, synovial sarcoma, and Ewing sarcoma. Demographic, tumor-specific, and treatment-specific factors were compared between tumors with an unknown grade and known grade using Pearson’s chi-square test. Multivariable logistic regression for the odds of having an unknown grade compared to a known grade adjusted for age, sex, race, histology, primary site, primary size, local extension, lymph node status, metastasis, surgical resection of the primary, and radiation therapy was performed. Further models were fit for the odds of undergoing surgery or radiation with grade as an independent predictor along with variables mentioned. Model fit was assessed with Akaike information criteria values and the discriminative ability of the model was evaluated using the concordance index, a generalization of the area under the receiver operating characteristic curve. Analyses were performed using Stata 14.1 for Windows (College Station, Texas) and SEER*Stat (Version 8.2.1). All tests of statistical significance were 2-sided with statistical significance established at α=0.05. The study was approved by the Institutional Review Board at Johns Hopkins University.

## CONCLUSIONS

In conclusion, this national study of sarcomas over 12 recent years highlights the increasing incidence of sarcomas and summarizes the epidemiology of sarcomas in the US. Conventional estimates of annual incidence of sarcomas appear to underestimate the true incidence by up to 50% by potentially excluding primary sites other than soft tissue. The substantial unknown information on grade in a nationally representative database and its association with lower utilization of surgery points to a lack of standardization in the diagnosis and treatment of sarcomas. Furthermore, the rise of frequency of sarcoma-NOS diagnosis is concerning for need of increased expertise in potentially complex cases. Regionalizing sarcoma care to specialized sarcoma centers equipped with a multi-disciplinary team who are dedicated to the care of these rare and heterogeneous tumors may ameliorate this trend of increasing inadequately graded, staged, and potentially treated sarcoma cases.

## SUPPLEMENTARY MATERIALS





## Abbreviations

US: United States
SEER: Surveillance, Epidemiology and End Results
NOS: not otherwise specified
MFH: malignant fibrous histiocytoma
GIST: gastrointestinal stromal tumor
CSM: cause specific mortality
ACM: all-cause mortality
ICD-O-3: International Classification for Oncology, 3^rd^ edition
WHO: World Health Organization
FNCLCC: French Federation of Cancer Centers Sarcoma Group
AJCC: American Joint Commission on Cancer
IQR: Interquartile Range
