# Supplementary material for: Sarcomas in the United States: Recent trends and a call for improved staging
Source: Oncotarget. 2019 Mar 29;10(25):2462–74. doi: 10.18632/oncotarget.26809 (PMC6497437; doi:10.18632/oncotarget.26809)
Supplement: Supplementary file 1 [file oncotarget-10-2462-s001.pdf]

# Sarcomas in the United States: Recent trends and a call for improved staging

## SUPPLEMENTARY MATERIALS

**Appendix A: Inclusion and exclusion using International Classification for Oncology, 3rd edition (ICD-O-3) histology codes**

See Supplementary File 1
