# Supplementary material for: Sarcomas in the United States: Recent trends and a call for improved staging
Source: Oncotarget. 2019 Mar 29;10(25):2462–74. doi: 10.18632/oncotarget.26809 (PMC6497437; doi:10.18632/oncotarget.26809)
Supplement: Supplementary file 2 [file oncotarget-10-2462-s002.docx]

Appendix A: Inclusion and exclusion using International Classification for Oncology, 3^rd^ edition (ICD-O-3) histology codes

| **Major histological groups** | **Included categories** | **ICD-O-3 codes** | |
| --- | --- | --- | --- |
| **Included histological types** |  |  |  |
| **Leiomyosarcoma** | Leiomyosarcoma NOS | 8890 - 8897 |  |
|  | Epitheloid | 8891 |  |
|  | Angiomyosarcoma | 8894 |  |
|  | Myosarcoma | 8895 |  |
|  | Myxoid Leiomyosarcoma | 8896 |  |
|  |  |  |  |
| **MFH** | MFH | 8830 |  |
|  |  |  |  |
| **Liposarcoma** | Liposarcoma NOS | 8850 |  |
|  | Well differentiated | 8851 |  |
|  | Myxoid Leiomyosarcoma | 8852 |  |
|  | Round cell | 8853 |  |
|  | Pleomorphic | 8854 |  |
|  | Mixed | 8855 |  |
|  | Fibroblastic | 8857 |  |
|  | Dedifferentiated | 8858 |  |
|  |  |  |  |
| **Dermatofibrosarcoma** | Dermatofibrosarcoma | 8832 |  |
|  |  |  |  |
| **Rhabdomyosarcoma** | Rhabdomyosarcoma NOS | 8900 |  |
|  | Pleomorphic | 8901 |  |
|  | Mixed type | 8902 |  |
|  | Embryonal | 8910 |  |
|  | Spindle | 8912 |  |
|  | Alveolar | 8920-8921 |  |
|  |  |  |  |
| **Angiosarcoma** | Hemangiosarcoma | 9120 |  |
|  | Kupffer cell sarcoma | 9124 |  |
|  | Hemangioendothelioma | 9130 |  |
|  | Epithelial Hemangioendothelioma | 9133 |  |
|  | Lymphangiosarcoma | 9170 |  |
|  |  |  |  |
| **Gastrointestinal stromal tumor (GIST)** | Gastrointestinal stromal sarcoma | 8936 |  |
|  |  |  |  |
| **Fibrosarcoma** | Fibrosarcoma NOS | 8810 |  |
|  | Fibromyxosarcoma | 8811 |  |
|  | Periosteal sarcoma NOS | 8812 |  |
|  | Fascial fibrosarcoma | 8813 |  |
|  | Infantile fibrosarcoma | 8814 |  |
|  | Solitary fibrous tumor | 8815 |  |
|  |  |  |  |
| **Sarcoma, NOS** | Sarcoma NOS | 8800 |  |
|  | Spindle cell sarcoma | 8801 |  |
|  | Giant cell | 8802 |  |
|  | Small cell | 8803 |  |
|  | Epitheliod cell | 8804 |  |
|  | Undifferentiated sarcoma | 8805 |  |
|  |  |  |  |
| **Osteosarcoma** | Osteosarcoma NOS | 9180 |  |
|  | Chondroblastic osteosarcoma | 9181 |  |
|  | Fibroblastic osteosarcoma | 9182 |  |
|  | Telangiectatic osteosarcoma | 9183 |  |
|  | Osteosarcoma in Paget | 9184 |  |
|  | Small cell | 9185 |  |
|  | Central osteosarcoma | 9186 |  |
|  | Intraosseous | 9187 |  |
|  | Parosteal osteosarcoma | 9192 |  |
|  | Periosteal osteosarcoma | 9193 |  |
|  | High grade surface osteosarcoma | 9194 |  |
|  | Intracorticol osteosarcoma | 9195 |  |
|  |  |  |  |
| **Chondrosarcoma** | Condrosarcoma NOS | 9220 |  |
|  | Juxtacortical chondrosarcoma | 9221 |  |
|  | Chondroblastoma | 9230 |  |
|  | Myxoid | 9231 |  |
|  | Mesenchymal | 9240 |  |
|  | Clear cell | 9242 |  |
|  | Dedifferentiated | 9243 |  |
|  |  |  |  |
| **Synovial sarcoma** | Synovial NOS | 9040 |  |
|  | Spindle cell | 9041 |  |
|  | Epitheloid cell | 9042 |  |
|  | Biphasic | 9043 |  |
|  |  |  |  |
| **Stromal sarcoma** | Endometrial stromal sarcoma | 8930-8931 |  |
|  | Adenosarcoma | 8933 |  |
|  | Carcinofibroma | 8934 |  |
|  | Stromal tumor NOS | 8935 |  |
|  |  |  |  |
| **Malignant peripheral nerve sheath tumors (MPNST)** | Malignant peripheral nerve sheath tumor | 9540 |  |
|  | Plexiform neurofibroma | 9550 |  |
|  | Malignant neurilemmoma | 9560 |  |
|  | MPNST with rhabdomyoblastic differentiation | 9561 |  |
|  | Malignant perineurioma | 9571 |  |
|  |  |  |  |
| **Ewing sarcoma** | Ewing's sarcoma | 9260 |  |
|  | Adamantinoma of long bones | 9261 |  |
|  |  |  |  |
| **Other** | Desmoplastic small cell tumor | 8806 |  |
|  | Myofibroblastic tumor | 8825 |  |
|  | Pigmented dermatofibrosarcoma protuberans | 8833 |  |
|  | Myxosarcoma | 8840 |  |
|  | Malignant mesenchymoma | 8990-8991 |  |
|  | Clear cell sarcoma NOS | 9044 |  |
|  | Malignant hemangiopericytoma | 9150 |  |
|  | Malignant giant cell tumor | 9250 |  |
|  | Malignant giant cell | 9251 |  |
|  | Malignant tenosynovial | 9252 |  |
|  | Malignant odontogenic tumor | 9270 |  |
|  | Malignant granular cell tumor | 9580 |  |
|  | Alveolar soft part sarcoma | 9581 |  |
| **Excluded histological types** |  |  |  |
|  | Germ cell tumors | 9060-9065 |  |
|  | Embryonic tumors | 9070-9073 |  |
|  | Teratoma | 9080-9085 |  |
|  | Choriocarcinoma | 9100-9105 |  |
|  | Phylloids tumor | 9020 |  |
|  | Carcinosarcoma | 8980-8982, 8183 |  |
|  | Brenner tumor | 9000 |  |
|  | Malignant mixed tumor | 8940-41 |  |
|  | Mesonephroma | 9110 |  |
|  | Stroma Ovarii | 9090-9091 |  |
|  | Mullerian mixed tumors | 8950, 8951, 8959 |  |
|  | Hepatoblastoma | 8970 |  |
|  | Pancreatoblastoma | 8971 |  |
|  | Pulmonary blastoma | 8972 |  |
|  | Plueropulmonary blastoma | 8973 |  |
|  | Nephroblastoma | 8960-8964 |  |
|  | Retinoblastoma | 9510-9513 |  |
|  | Hemangioblastoma | 9161 |  |
|  | Adenocarcinofibroma | 9014-9015 |  |
|  | Kaposi's sarcoma | 9140-9143 |  |
